# Oat Intake and Risk of Type 2 Diabetes, Cardiovascular Disease and All-Cause Mortality: A Systematic Review and Meta-Analysis

**DOI:** 10.3390/nu13082560

**Published:** 2021-07-26

**Authors:** Faina Wehrli, Petek E. Taneri, Arjola Bano, Lia Bally, Lauren C. Blekkenhorst, Weston Bussler, Brandon Metzger, Beatrice Minder, Marija Glisic, Taulant Muka, Hua Kern

**Affiliations:** 1 Institute of Social and Preventive Medicine (ISPM), University of Bern, 3012 Bern, Switzerland; faina.wehrli@ispm.unibe.ch (F.W.); eyltnr@gmail.com (P.E.T.); arjola.bano@ispm.unibe.ch (A.B.); marija.glisic@ispm.unibe.ch (M.G.); 2 Department Istanbul, Faculty of Medicine Public Health, Bahcesehir University, Istanbul 34734, Turkey; 3 Department of Cardiology, Inselspital, Bern University Hospital, University of Bern, 3010 Bern, Switzerland; 4 Department of Diabetes, Endocrinology, Nutritional Medicine, and Metabolism, Inselspital, University of Bern, 3010 Bern, Switzerland; Lia.Bally@insel.ch; 5 School of Medical and Health Sciences, Edith Cowan University, Joondalup, WA 6027, Australia; l.blekkenhorst@ecu.edu.au; 6 Medical School, The University of Western Australia, Perth, WA 6000, Australia; 7 Standard Process Nutrition Innovation Center, Kannapolis, NC 28018, USA; wbussler@Standardprocess.com (W.B.); bmetzger@Standardprocess.com (B.M.); ckern@Standardprocess.com (H.K.); 8 Public Health & Primary Care Library, University Library of Bern, University of Bern, 3012 Bern, Switzerland; beatrice.minder@ispm.unibe.ch

**Keywords:** oat, type 2 diabetes, cardiovascular disease, mortality

## Abstract

Cardiovascular disease (CVD) and type 2 diabetes (T2D) remain the top disease and mortality burdens worldwide. Oats have been shown to benefit cardiovascular health and improve insulin resistance. However, the evidence linking oat consumption with CVD, T2D and all-cause mortality remains inconclusive. We conducted a comprehensive systematic review and meta-analysis of prospective cohort studies to evaluate the associations between oat consumption and risks of T2D, CVD and all-cause mortality in the general population. Five electronic databases were searched until September, 2020. Study specific relative risks (RR) were meta-analyzed using random effect models. Of 4686 relevant references, we included 9 articles, based on 8 unique studies and 471,157 participants. Comparing oat consumers versus non-consumers, RRs were 0.86 (95% CI 0.72–1.03) for T2D incidence and 0.73 (95% CI 0.5–1.07) for combined CVD incidence. Comparing participants with highest versus lowest oat intake, RRs were 0.78 (95% CI 0.74–0.82) for T2D incidence, 0.81 (95% CI 0.61–1.08) for CHD incidence and 0.79 (95% CI 0.59–1.07) for stroke. For all-cause mortality one study based on three cohorts found RR for men and women were 0.76 (95% CI 0.69–0.85) and 0.78 (95% CI 0.70–0.87), respectively. Most studies (*n* = 6) were of fair to good quality. This meta-analysis suggests that consumption of oat could reduce the risk for T2D and all-cause mortality, while no significant association was found for CVD. Future studies should address a lack of standardized methods in assessing overall oat intake and type of oat products, and investigate a dose-dependent response of oat products on cardiometabolic outcomes in order to introduce oat as preventive and treatment options for the public.

## 1. Introduction

Oats (Avena sativa) are a whole-grain cereal predominantly grown in Europe and the Americas [1]. They are recognized for their fiber content, especially beta-glucan, and are a rich source of many vitamins and minerals, and have the highest protein content among cereals and other bioactive compounds [2]. Whole oats contain over 20 unique polyphenols, such as avenanthramides, which have demonstrated strong antioxidant activity in vivo and in vitro [3]. Due to their potential health benefits, oat consumption is increasing in the western world. Although oatmeal (thinly cut whole grain oats) is consumed worldwide, the primary markets are North America and North Europe [1]. Carl von Noorden, a German physician and diabetologist, reported the first therapeutic effects of oats—the attenuation of blood glucose levels—in 1903 [4]. Since then, oats have been shown to improve insulin sensitivity [5], glucose metabolism [6,7,8], blood lipid profile [9], endothelial function [10] and inflammation [11], all important markers associated with the development of type 2 diabetes (T2D), cardiovascular disease (CVD) and all-cause mortality. However, the association of oat intake with cardiometabolic disease and all-cause mortality in longitudinal observational studies remains inconsistent. Several studies have found inverse associations between oat consumption and impaired glucose metabolism in T2D [8,12], markers of CVD [13] and all-cause mortality [14], while others have reported no associations [15,16].

To find a consensus, a comprehensive analysis of the literature is needed. We, therefore, undertook a systematic review and meta-analysis of prospective studies to explore the associations of oat consumption with the risk of T2D, CVD and all-cause mortality in the general population.

## 2. Materials and Methods

We performed a systematic review and meta-analysis in accordance with a recently published guideline on conducting a systematic review and meta-analysis, as well as the PRISMA guidelines [17,18]. The protocol for the current study is registered in PROSPERO (ID:CRD42020196084).

### 2.1. Data Source and Strategy

We searched MEDLINE via Ovid, EMBASE, Web of Science Core Collection, Cochrane Library, and Google Scholar to identify relevant articles. Search terms included subject headings and free text words related to oat consumption (oat, oat bran, oatmeal, porridge, oat cereal, oat bran beta-glucan, oat whole grains) and adverse outcomes including incident CVD (coronary heart disease, heart failure, coronary artery disease, myocardial ischemia, stroke, myocardial infarction, cardiovascular/coronary heart disease death, fatal stroke), incident T2D and all-cause mortality. We searched the databases from inception until 18th September 2020. We limited our search to human studies. No limitations on publication date or language were used. We additionally searched the references of eligible articles and studies that have cited those articles. The complete search strategy is outlined in the Appendix A.

### 2.2. Study Selection and Eligibility Criteria

Studies were included if they (i) were of prospective design (nested case-control studies, case-cohort studies, prospective cohort studies and clinical trials); (ii) had reported on oat intake as part of the diet or as supplements, and (iii) had provided information on the association between oat intake/supplementation with risk of T2D, CVD and/or all-cause mortality. We excluded abstracts, cost-effectiveness studies, letters to the editor, conference proceedings, cross-sectional studies, case-control studies other than those of prospective design (e.g., nested case-control studies), systematic reviews, and meta-analyses. Studies, among adults, examining the association between oat consumption (not overall wheat) and outcomes of interest were included.

### 2.3. Data Extraction

The titles and abstracts were screened by two independent reviewers in accordance with the selection criteria (FW and PET). A data extraction form was used to record information such as the author’s name, study location, study design, sample size, baseline age, follow-up time, methods used to assess oat intake, outcome definition, number of events, measures of associations, and level of adjustment. Before beginning the full data extraction, the form was developed, piloted, and discussed within the review group. When multiple publications were assessed, the most recent or comprehensive information was used. Extracted data are summarized in Tables S2–S4.

### 2.4. Risk of Bias Assessment

The quality of included studies was independently assessed by two authors (FW and PET) using the Newcastle–Ottawa Scale for cohort studies [19]; a third author (TM) adjudicated if consensus could not be reached. The scale was developed for non-randomized and observational studies and assesses quality in three broad categories: study group/participant selection, group/participant comparability, and the assessment of exposure/outcome of interest. Quality was graded on a 10-point scale and classified as good (8–10 points), fair (5–7), or poor (<5). In addition, we used the Grading of Recommendations Assessment, Development and Evaluation (GRADE) method to assess the quality of evidence in the current review. The GRADE method evaluates evidence based on two key concepts: magnitude of effect and quality of evidence (considering the risk of bias, study design, consistency and directness of findings). The evidence is categorized as either high, moderate, low or very low. RCTs begin with high quality, whereas observational studies begin with low quality. Study limitations, significant inconsistency of results, or uncertainty about the directness of the evidence can all lower the grade of evidence. Furthermore, evidence of a dose response gradient or strong evidence of association based on consistent evidence from two or more observational studies with no plausible confounders may raise the grade [20]. Two reviewers worked independently on the evaluation, and any disagreements were resolved through discussion between the two parties or by bringing in a third reviewer.

### 2.5. Data Synthesis and Analysis

We calculated pooled relative risks (RR) and 95% confidence intervals (CI) for incident CVD, incident T2D, and all-cause mortality between oat consumers and non-consumers, and highest vs. lowest oat intake based on the extracted data from each study. Odds ratios reported by Xu X. et al., 2019 were converted to relative risks using the method described by Grant R, 2014 [21].

The inverse variance weighted method was used to combine RR to produce a pooled RR using random-effects models to account for between-study heterogeneity; as a sensitivity analysis, we reported the estimates derived from fixed effect models. Fixed effect models, on the other hand, were used to pool results from different groups in the same study, which were then included in our meta-analyses. For meta-analyses including 4 or more studies, we also assessed publication bias by funnel plot, and Egger’s test was used to assess asymmetry. All analyses were conducted with STATA 16.1 (StataCorp. 2019. College Station, TX, USA: StataCorp LLC.). We calculated 2-tailed tests and a *p*-value <0.05 was considered significant.

## 3. Results

### 3.1. Study Identification and Selection

**Figure 1:**
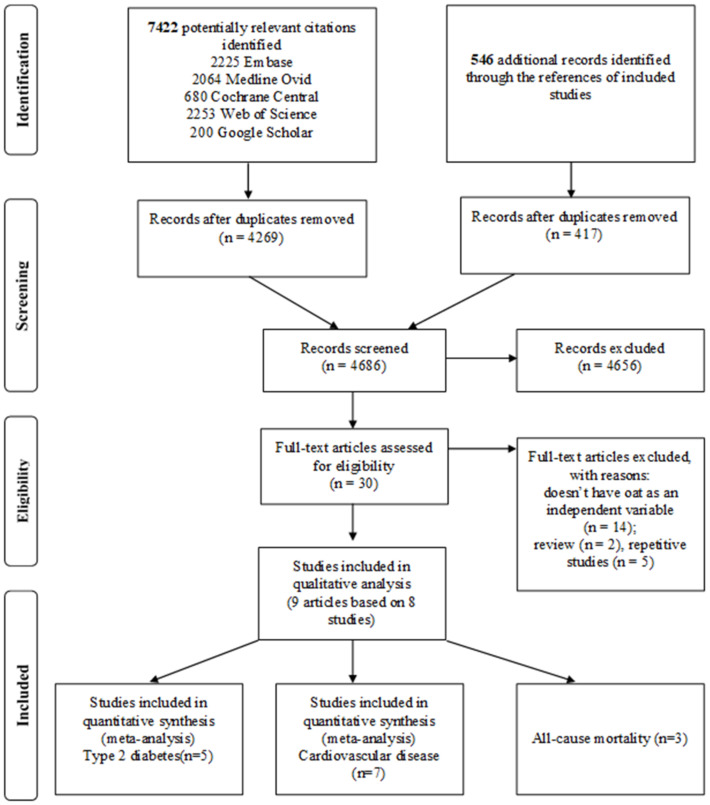
PRISMA flow diagram of search strategy.

A total of 4686 citations were identified, of which 30 were chosen for full-text evaluation (Figure 1). Of those, 9 articles based on a total of 8 unique studies reporting on 471,157 participants were included with relevant available data on T2D (5 studies), CVD (7 studies), and all-cause mortality (3 studies). Table 1 summarizes the detailed characteristics of the included studies. Of these, 3 were based in North America, 3 in Europe, and 2 in the Asia-Pacific region. All studies were prospective cohort studies. The follow up time ranged between 2.2 and 30 years. Most studies (*n* = 6) were of fair to good quality (score ≥ 7), while 2 studies were of poor quality (score < 5). The assessed oat intake varied between the studies: 3 studies analyzed oatmeal consumption, 1 investigated oatmeal and whole grain oat intake, 1 article assessed the effects of oat fiber from various oat containing products [22], 1 study analyzed specifically oat cereals [23], and 2 looked only at whole grain oats [24]. No study had reported investigating associations with oat extracts. Table S1 contains information on the specific characteristics of the studies that were included.

### 3.2. Association between Oat Consumption and Risk of Type 2 Diabetes

Five studies [23,26,28,29] were included in the meta-analysis of oat consumption and incidence of type 2 diabetes (Table 1). We previously excluded one article, since it was based on the Danish Diet, Cancer and Health cohort and the analysis was done earlier and on a smaller number of participants than another study [31] (Table S2). The studies included a total of 31,329 incident cases of T2D. The total duration of follow-up ranged from 6 to 30 years. All studies excluded patients with T2D diagnosis at baseline and adjusted for age, physical activity, smoking, alcohol consumption and body mass index. Three studies additionally adjusted for total energy intake; two also adjusted for use of multivitamins, family history of diabetes, educational level, red meat consumption and postmenopausal hormone use for women. One study also adjusted for dietary fiber consumption, ethnicity and marital status.

**Figure 2:**
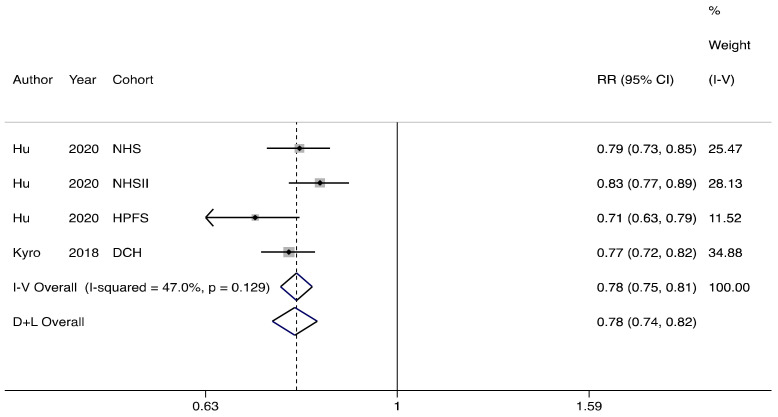
The association between oat consumption and risk of type 2 diabetes. Participants with highest vs. lowest oat consumption are compared. I-V: inverse-variance estimation, random effect model. D + L: DerSimonian–Laird (DL) method, fixed effect model [32].

Higher oat intake (more than 5.7 g/day) was significantly associated with lower risk of T2D when compared to lower consumption (less than 1.3g/day), with a relative risk of 0.78 (95% confidence interval 0.74 to 0.82; I^2^ = 47%, *p* = 0.129) (Figure 2).

**Figure 3:**
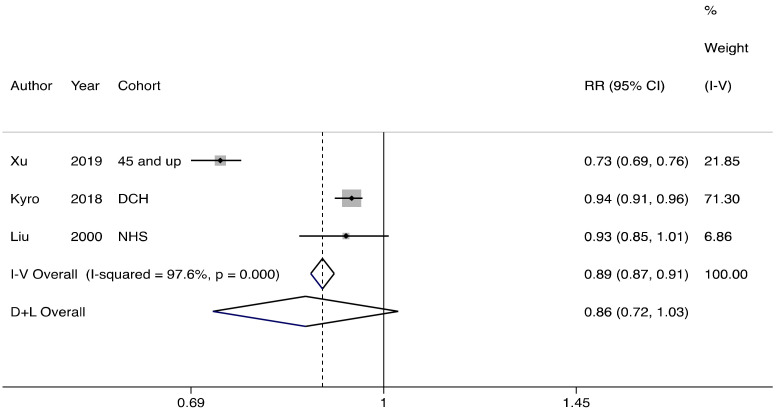
The association between oat consumption and risk of type 2 diabetes. Consumers vs. non-consumers are compared. I-V: inverse-variance estimation, random effect model. D + L: DerSimonian–Laird (DL) method, fixed effect model [32].

Dietary intake of oats was associated with a lower risk of T2D with a relative risk of 0.86, albeit not statistically significant (95% confidence interval 0.72 to 1.03; I^2^ = 97.6%, *p* < 0.001) (Figure 3). There was evidence of heterogeneity in T2D estimates across studies for oat consumption. Two studies using data from the Danish Diet, Cancer and Health cohort reported that men and women consuming >21 g/day of oatmeal/muesli had a lower risk of developing T2D (21–27% decreased risk in the more recent study) (data from [28]).

### 3.3. Association between Oat Consumption and Risk of Cardiovascular Disease

Seven studies [22,23,24,25,27,30] were included in the meta-analysis of oat consumption and risk of cardiovascular disease. Three articles, including the Danish Diet, Cancer, and Health (*n* = 1) cohort and the Nurses’ Health Study cohort (*n* = 2), were excluded since new studies on the same cohorts analyzing the same outcomes were included in the analysis (Table S2). The meta-analysis included 18,128 cases of cardiovascular disease, coronary heart disease, myocardial infarction, or stroke from seven contributing studies. The total duration of follow-up ranged from 6 to 26 years. All included studies adjusted for age and smoking, six studies additionally adjusted for alcohol consumption, physical activity and body mass index, four studies additionally adjusted for education and hypertension, and three for cholesterol, total energy intake and menopausal status and hormone therapy for women. Two studies additionally adjusted for gender, ethnicity and diabetes history.

**Figure 4:**
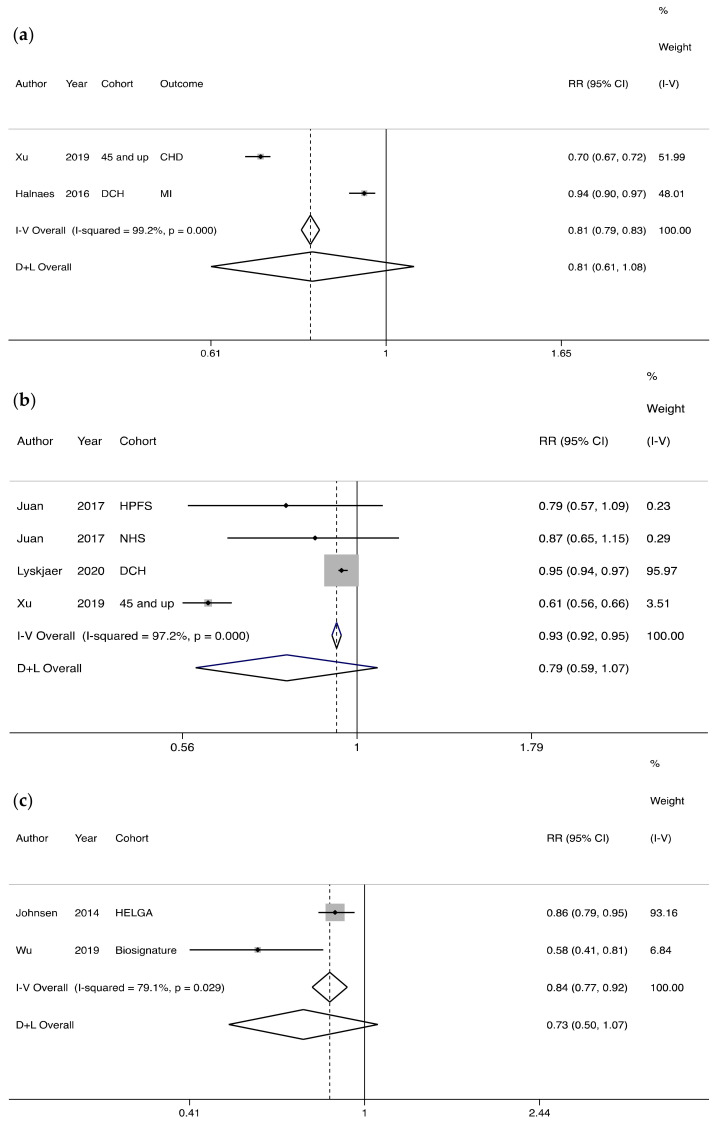
The association between oat consumption and risk of cardiovascular disease. (**a**). The association between oat consumption and risk of cardiovascular disease. Participants with highest vs. lowest oat consumption are compared. CHD–coronary heart disease, MI–myocardial infraction. (**b**). The association between oat consumption and risk of stroke. Participants with highest vs. lowest oat consumption are compared. (**c**). The association between oat consumption and risk of composite cardiovascular disease. Consumers vs. non-consumers are compared. I–V: inverse-variance estimation, random effect model. D + L: DerSimonian–Laird (DL) method, fixed effect model [32].

No significant associations were found between oat intake as a continuous dietary exposure and risk of coronary heart disease and myocardial infarction combined together, or stroke with relative risks of 0.81 (95% confidence interval 0.61 to 1.08; I^2^ = 99.2%, *p* = 0.000) and 0.79 (95% confidence interval 0.59 to 1.07; I^2^ = 97.2%, *p* = 0.000), respectively (Figure 4a,b). Dietary intake of oats was also not associated with the risk of composite cardiovascular diseases (relative risk 0.73, 95% confidence interval 0.5 to 1.07; I^2^ = 78.9%, *p* = 0.029; Figure 4c). There was evidence of heterogeneity in cardiovascular disease estimates across studies for oat consumption.

### 3.4. Association between Oat Consumption and All-Cause Mortality

We found two articles reporting the effects of oats on all-cause mortality, with an overlapping cohort (Danish Diet, Cancer, and Health cohort; [14,24]). We included the most recent study in our analysis. In total, one article describing three individual cohorts investigated the association between oat intake and all-cause mortality, with 7839 cases of all-cause mortality. The median follow-up duration ranged from 11.1 to 14.2 years in these prospective studies. The study adjusted for age, follow up time, education, smoking intensity, alcohol intake, BMI and total energy intake. When comparing participants with higher vs. lowest oat intake, both men and women with highest consumption (>19 g/day) had significantly lower risks for all-cause mortality with a relative risk of 0.76 (95% confidence interval 0.69 to 0.85) and 0.78 (95% confidence interval 0.7 to 0.87), respectively.

### 3.5. Study Quality

**Figure 5:**
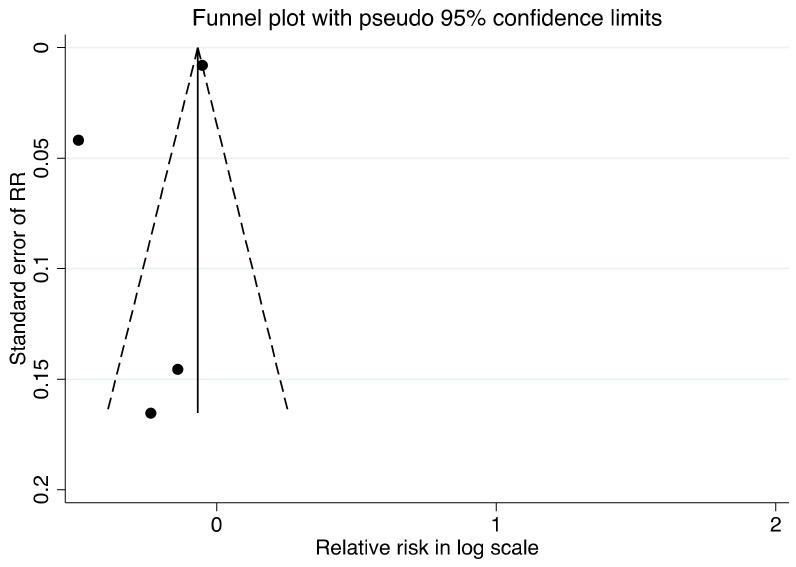
Funnel plot for association of oat intake with cardiovascular disease.

For risk of T2D and CVD, the results were of low and very low certainty. The evidence was based solely on the observational data. Despite the fact that the study population and each outcome showed good generalizability, we found some heterogeneity. We were unable to investigate the publication bias for other outcomes due to the small number of studies. There was no evidence of publication bias in the study of oat intake and CVD risk (*p* > 0.05, Figure 5). Imprecision, inconsistency and risk of bias were a problem for most subgroups. Table S5 summarizes the assessment of evidence quality.

### 3.6. Sensitivity Analysis

The fixed effects models showed significant associations of oat intake (high vs. low) with incidence of type 2 diabetes, composite cardiovascular disease and stroke ( Figure 2; Figure 4a,b). Under the fixed effects model, compared to non-consumers, oat consumers had a lower risk of developing risk of T2D, but not of cardiovascular disease ( Figure 3; Figure 4c).

## 4. Discussion

### 4.1. Principal Findings

We conducted a systematic review and meta-analysis using data from approximately 471,157 participants from 8 studies to help clarify available evidence on the associations of oat intake with the risk of type 2 diabetes, cardiovascular disease, and all-cause mortality. Overall, our findings show that a high oat consumption is associated with a lower risk of T2D and all-cause mortality, but not consistently with CVD.

### 4.2. Comparison with Other Studies

To date, this is the first meta-analysis to investigate the association between oat intake and risk of type 2 diabetes, cardiovascular disease and all-cause mortality. Our findings are in agreement with multiple published meta-analyses, reporting positive effects of oat intake on T2D risk factors, such as lowering HbA1c, fasting and postprandial glucose and fasting insulin [5]. Since oats are considered to be a whole grain, our findings are consistent with the previously published data on beneficial effects of whole grains on the risk of coronary heart disease, CVD, total cancer, and mortality from all causes [33]. Moreover, whole grain oats appeared to be the most effective whole grain in terms of cholesterol reduction [34].

### 4.3. Potential Underlying Mechanisms

Oats deliver high amounts of valuable nutrients, including proteins, minerals, B vitamins, and iron, but a substantial amount of the beneficial effect is attributed to the fiber content. Fiber intake in general has been found to be associated with reduced risks of all-cause mortality, CVD and all cancers [35]. In multiple randomized controlled trials, fiber consumption decreased LDL cholesterol [36], postprandial glucose and insulin [37], while some meta-analyses have reported inverse associations between fiber and risk of metabolic syndrome [38], decrease in BMI, body weight, fasting glucose, fasting insulin [39] and lower systolic and diastolic blood pressure [40], all risk factors for type 2 diabetes and cardiovascular disease. The principal component of oat fiber is beta-glucan, a non-digestible polysaccharide, that cannot be absorbed in the small intestine. Beta-glucan is water-soluble and increases the viscosity of the alimentary bolus in the upper gastrointestinal tract, slowing nutrient absorption and thus postprandial glucose excursions [41]. Specifically, beta-glucan intake from oats has been associated with lower total and LDL cholesterol [9], lower appetite [42] and higher serum nitric oxide, an important cell signaling molecule essential for vascular health and lowering blood pressure [43]. Avenanthramides, a unique type of phenolic compounds present in oats with antioxidant and anti-inflammatory properties, have also been reported to increase nitric oxide bioavailability and hence lower blood pressure [10,44]. In addition, a number of clinical trials have also found that oats improve glucose control [6,7,8]. Whole oats deliver many bioactive compounds simultaneously and have shown superior ability to help manage glucose control and insulin sensitivity when compared to isolated beta-glucans from oats [45]. Oats also have a high satiety index, together with the releasing effect of the anorexigenic peptide YY by beta-glucan, which can lead to lower caloric intake and may decrease the risk of obesity [46]. In addition, low calorie diets can have positive effects on diabetes [47] and longevity [48]. Two recently completed clinical trials might shed more light on the mechanism of oat beta-glucan (NCT04299763), oat bran (NCT03805802) and oat powder (NCT03911427) effects on T2D and CVD risk factors.

### 4.4. Strengths and Limitations of the Study

This work’s strengths and weaknesses deserve careful consideration. This is the first comprehensive meta-analysis using a predefined protocol, investigating the associations of oat consumption with risk of T2D, CVD and all-cause mortality. The quality of included studies was good to moderate and the numbers of participants and the analyzed events were high. Several limitations also warrant mentioning. Because only 9 articles met our search criteria, we may not have enough power to detect a definite effect in the case of cardiovascular disease risk. Due to the limited number of studies included in each analysis, it was also not possible to define the sources of heterogeneity observed in our meta-analyses. The possible reasons for the null association with CVD could be the difference in assessed oat intake in different populations: 8 articles analyzed oatmeal consumption, 3 assessed oatmeal and whole grain oat intake, one article assessed the effects of oat fiber from various oat containing products [22], one study analyzed oat cereals specifically [23], and one looked only at whole grain oats [24], as well as different questionnaires used for consumption assessment. Median oat intake also tended to vary between different studies: from median 0.7 g/day [30] to 2 g/day [24]. The follow-up period of one of the studies was only 6 years, which might not be long enough for the development of cardiovascular disease. The results for T2D are mainly based on three large cohort studies conducted in health professionals in the US, while the results were statistically significant, future studies analysing effects of oats in other countries/other settings will be beneficial. Because the available data for the meta-analysis on oat intake were rather limited, heterogeneity across studies was high, even though we used random-effects, and studies of oat intake affecting all-cause mortality were few. Therefore, future large-scale studies would allow for a more detailed and specific assessment of the relationship between the oat and T2D, CVD, and all-cause mortality, such as dosage assessment and standardized adjustment for confounders. (i.e., smoking status, BMI, quality of diet), evaluation of heterogeneity among diverse study populations. We were only able to conduct analysis on consumers vs. non-consumers and participants with high vs. low oat intake due to the limited number of eligible studies. We were not able to address the question of at what dosage the beneficial effects of oat consumption would trigger the positive outcome. Future studies should explore whether the association between oat and health outcomes depends on the quantity of oat consumption. Moreover, since there was only one article based on three studies which we found eligible to assess the effect of oats on all-cause mortality, we could not perform a meta-analysis to address this topic. The cohorts in this study are all from developed Scandinavian counties, and while the findings are promising, more studies from other counties are required to determine if the results are reproducible. GRADE assessment indicated that further high-quality randomized trials are needed for a firm conclusion.

### 4.5. Implications for Clinicians and Policy Makers

Our findings could have significant policy and scientific ramifications. These findings emphasize the importance of including oats and/or oat products in the diet to reduce the risk of T2D and possibly CVD (as suggested by the results of fixed effects models) two of the most common noncommunicable diseases worldwide. Cardiovascular diseases together with type 2 diabetes cause 19.5 million deaths annually (WHO noncommunicable disease report 2018). Most of these deaths are premature and could be prevented by educating health care providers as well as the public about the benefits of a healthy life style such as a healthy diet. Given that current global noncommunicable disease prevention strategies (e.g., WHO Global Action Plan 2013–2020) recommend an increase in fruit and vegetable consumption, our findings may have important policy implications. Despite recommendations from several health organizations to increase consumption of fiber-rich foods, fiber intake worldwide remains well below recommended levels [41]. Recognizing oats as an additional source of fiber will thus help gain wider socio-political support for establishing appropriate legislation, preventive strategies, standards, and public recommendations to combat these major global noncommunicable diseases. Fiber inclusion in traditional and processed foods is one way to accomplish this [49]. The food industry has aimed to develop new products geared toward functional foods and ingredients in response to consumer demand for healthier options. Oat and oat beta-glucan could be incorporated into breakfasts, baking products, milk and meat alternatives [49]. Oats were reported not only for prevention but also for the treatment of T2D. Short term oat interventions were shown to decrease blood glucose concentrations and to improve insulin sensitivity in patients as early as in 1903 [4]. In series of clinical trials, introduction of low-fat plant-based diets for patients with type 2 diabetes was shown to improve glycemic control [50]. Therefore, it would be beneficial to conduct further larger population studies with adequate follow-up duration, investigating standardized oat intake in a dose-dependent manner, and include oats in the future as a necessary component of prevention and treatment options for type 2 diabetes and cardiovascular diseases. Additionally, not all oats are equal in their concentration of health beneficial compounds or biological effects [51]. Plant breeding efforts and oats selected for their increased concentration of health beneficial components, or demonstrated beneficial effects can be supported.

## 5. Conclusions

Results of this meta-analysis indicate a potential beneficial role of oat consumption in type 2 diabetes and mortality. However, the evidence is limited and mainly from observational data, making it difficult to draw firm conclusions. Further detailed work with large studies and clinical trials is needed to better characterize these associations and to assess causality.

## Figures and Tables

**Table 1:** Summary of the studies included in the systematic review.

Reference	Study Location	Cohort Name	No. of Individuals	Median Age (5th–95th Percentile)	Percentage of Males	Mean/Medium Follow up Time, Years	Oat Intake	Median Oat Intake in Population, g/day	Number of Events			Study Quality
									T2D	CVD	All-Cause Mortality	
Helnaes (2016) [25]	Denmark	Danish Diet, Cancer, and Health cohort (DCH)	54871	all m ^1^: 25921: 55.0 (50.0–64.0)	47.2	13.6 median	oatmeal	1	NA	2329	NA	8
				cases m: 1676: 57.0 (51.0–64.0)								
				all w ^1^: 28950: 56.0 (50.0–64.0)								
				cases w: 653: 59.0 (51.0–64.0)								
Hu (2020) [26]	US	Nurses’ Health Study (NHS)	69139	mean 30	0	24 mean	oatmeal	NA	9723	NA	NA	7
		Nurses’ Health Study II (NHSII)	89120		0				6821			
		Health Professionals Follow-up Study (HPFS)	36525		100				2085			
Johnsen (2015) [24]	Scandinavian	HELGA ^2^	119518	w: 51 (40–63) m: 54 (31–64)	33	NA	whole grain oats	2	NA	298 women, 858 men died from CHD ^3^, 137 women, 143 men dies from stroke	7839	7
	Norway	the Norwegian Women and Cancer Study	37111		0	11.1 median			NA		966	
	Sweden	the Vasterbotten Intervention Programme cohort	25551		48.3	14.2 median			NA		1367	
	Denmark	the Danish Diet Cancer and Health Study	56865		47.6	11.9 median			NA		5506	
Juan (2017) [27]	US	Health Professionals Follow-up Study	42823	mean (SD) 53.2(9.5)	100	24	oatmeal	NA	NA	NA	908	7
		Nurses’ Health Study	71750	mean (SD) 50.1(7.1)	0	26					1550	
Kyro (2018) [28]	Denmark	Diet, Cancer, and Health cohort	55465	50–65	47.3	15	whole grain oat, oatmeal	1	7417	NA	NA	9
Liu (2000) [29]	US	Nurses’ Health Study	75521	38–63 mean	0	10	oatmeal	NA	1879	NA	NA	7
Lyskjaer (2020) [30]	Denmark	Diet, Cancer, and Health cohort	55095	56.1(52.7–60.3)	47.6	13.4 median	oatmeal	0.7	NA	2260	NA	8
Olsen (2011) [14]	Denmark	Diet, Cancer, and Health cohort	57053	50–64	46.3	12	oatmeal	0.8	NA	NA	4126	9
Xu (2019) [23]	Australia	45 and Up Study	142503	45–64y 37,626 all cases	NA	3	oat cereal	NA	5283	Heart disease 14148, stroke 2911	NA	5
				65–80y 24,203 all cases								
				>80 y 5967								
Wu (2019) [22]	Taiwan	The Biosignature study	1663	mean ± SD nouse:65.26 ± 12.19 oat:68.82 ± 11.65	36.6	mean 26.75 ± 8.11 months	oat fiber (oat bran, oat containing products)	NA	NA	175	N/A	6

^1^ m–number of men and w–number of women in the study. ^2^ HELGA cohort consists of The Norwegian Women and Cancer Study, The Northern Sweden Health and Disease Study and The Danish Diet, Cancer and Health Cohort Study. ^3^ CHD–coronary heart disease.

## Supplementary Materials

The following are available online at https://www.mdpi.com/article/10.3390/nu13082560/s1, Table S1: Summary of the studies included in the systematic review; Table S2: Extraction table for incidence of T2D; Table S3: Extraction table for incidence of CVD. A). Continuous exposure; B). Categorical exposure; Table S4: Extraction table for all-cause mortality; Table S5: GRADE assessment. Does oat consumption influence the risk of T2D and CVD in the general population.

## Appendix A. Full Search Strategy per Database

**Embase.com**

(‘oat’/de OR ‘oat bran’/de OR ‘beta glucan’/de OR ‘whole grain’/de OR (‘avena sativa’ OR oat OR oats OR oatmeal* OR oatcake* OR porridge* OR muesli OR granola OR b-glucan* OR β-glucan* OR beta-glucan* OR beta-dextroglucan* OR ‘whole grain*’ OR wholegrain*):ab,ti,kw) AND ((‘cardiovascular disease’/de OR ‘cardiometabolic disease’/exp OR ‘heart failure’/de OR ‘congestive heart failure’/de OR ‘heart disease’/de OR ‘hypertensive heart disease’/exp OR ‘hypertensive heart failure’/exp OR ‘coronary artery disease’/de OR ‘ischemic heart disease’/exp OR ‘cerebrovascular accident’/de OR ‘cerebral artery disease’/de OR ‘atherosclerotic cardiovascular disease’/de OR ‘brain ischemia’/exp OR ‘cardiovascular mortality’/de OR ‘mortality’/de OR (((cardiovascular OR coronar* OR cardiac OR heart OR myocard* OR cardiometabol* OR cardio-metabol*) NEAR/3 (disease* OR event* OR infarct* OR disorder* OR function* OR dysfunction* OR health OR mortalit*)) OR cvd OR cvds OR cardiopath* OR angina OR ((vascular OR ‘peripheral arter*’) NEAR/2 disease*) OR ((ischemi* OR ischaemi* OR fail* OR attack* OR insufficien*) NEAR/3 (heart OR cardia* OR myocard*)) OR (cerebrovascular* NEAR/3 accident*) OR cva OR stroke* OR ((brain OR cerebral) NEAR/3 (ischemi* OR ischaemi*)) OR mortalit*):ab,ti,kw) OR (‘non insulin dependent diabetes mellitus’/exp OR (((diabet* OR dm) NEAR/3 (‘type 2’ OR type2 OR ‘type ii’ OR ‘non insulin’ OR noninsulin OR ‘adult onset’ OR ‘slow onset’ OR ‘maturity onset’)) OR T2DM OR dmt2 OR dm2 OR T2-DM OR dm-t2 OR dm-2 OR niddm OR nid-dm OR MODY):ab,ti,kw)) NOT ([animals]/lim NOT [humans]/lim) NOT ([Conference Abstract]/lim OR [Letter]/lim OR [Note]/lim OR [Editorial]/lim)

**Medline (Ovid)**

(Avena/OR exp beta-Glucans/OR Whole Grains/OR (avena sativa OR oat OR oats OR oatmeal* OR oatcake* OR porridge* OR muesli OR granola OR b-glucan* OR beta-glucan* OR beta-dextroglucan* OR whole grain* OR wholegrain*).ab,ti,kw.) AND ((“Cardiovascular Diseases”/OR exp “Heart Failure”/OR “Heart Diseases”/OR exp “Coronary Artery Disease”/OR exp “Myocardial Ischemia”/OR exp “Stroke”/OR “Atherosclerosis”/OR exp “Brain Ischemia”/OR exp Cerebral Arterial Diseases/OR Mortality/OR mortality.fs. OR (((cardiovascular OR coronar* OR cardiac OR heart OR myocard* OR cardiometabol* OR cardio-metabol*) ADJ3 (disease* OR event* OR infarct* OR disorder* OR function* OR dysfunction* OR health OR mortalit*)) OR cvd OR cvds OR cardiopath* OR angina OR ((vascular OR peripheral arter*) ADJ2 disease*) OR ((ischemi* OR ischaemi* OR fail* OR attack* OR insufficien*) ADJ3 (heart OR cardia* OR myocard*)) OR (cerebrovascular* ADJ3 accident*) OR cva OR stroke* OR ((brain OR cerebral) ADJ3 (ischemi* OR ischaemi*)) OR mortalit*).ab,ti,kw.) OR (“Diabetes Mellitus, Type 2”/OR (((diabet* OR dm) ADJ3 (“type 2” OR type2 OR “type ii” OR “non insulin” OR noninsulin OR “adult onset” OR “slow onset” OR “maturity onset”)) OR T2DM OR dmt2 OR dm2 OR T2-DM OR dm-t2 OR dm-2 OR niddm OR nid-dm OR MODY).ab,ti,kw.)) NOT (exp animals/NOT humans/) NOT (letter* OR news OR comment* OR editorial* OR congres* OR abstract* OR book* OR chapter* OR dissertation abstract*).pt.

**Cochrane Library (Wiley)**

((‘avena sativa’ OR oat OR oats OR oatmeal* OR oatcake* OR porridge* OR muesli OR granola OR b-glucan* OR β-glucan* OR beta-glucan* OR beta-dextroglucan* OR ‘whole grain*’ OR (whole NEXT grain*) OR wholegrain*):ab,ti,kw) AND ((((cardiovascular OR coronar* OR cardiac OR heart OR myocard* OR cardiometabol* OR cardio-metabol*) NEAR/3 (disease* OR event* OR infarct* OR disorder* OR function* OR dysfunction* OR health OR mortalit*)) OR cvd OR cvds OR cardiopath* OR angina OR ((vascular OR peripheral NEXT arter*) NEAR/2 disease*) OR ((ischemi* OR ischaemi* OR fail* OR attack* OR insufficien*) NEAR/3 (heart OR cardia* OR myocard*)) OR (cerebrovascular* NEAR/3 accident*) OR cva OR stroke* OR ((brain OR cerebral) NEAR/3 (ischemi* OR ischaemi*)) OR mortalit*):ab,ti,kw OR (((diabet* OR dm) NEAR/3 (‘type 2’ OR type2 OR ‘type ii’ OR ‘non insulin’ OR noninsulin OR ‘adult onset’ OR ‘slow onset’ OR ‘maturity onset’)) OR T2DM OR dmt2 OR dm2 OR “T2-DM” OR “dm-t2” OR “dm-2” OR niddm OR “nid-dm” OR MODY):ab,ti,kw)

**Web of Science Core Collection**

TS = ((((“avena sativa” OR oat OR oats OR oatmeal* OR oatcake* OR porridge* OR muesli OR granola OR b-glucan* OR β-glucan* OR beta- glucan* OR beta-dextroglucan* OR “whole grain*” OR wholegrain*)) AND ((((cardiovascular OR coronar* OR cardiac OR heart OR myocard* OR cardiometabol* OR cardio-metabol*) NEAR/3 (disease* OR event* OR infarct* OR disorder* OR function* OR dysfunction* OR health OR mortalit*)) OR cvd OR cvds OR cardiopath* OR angina OR ((vascular OR “peripheral arter*”) NEAR/2 disease*) OR ((ischemi* OR ischaemi* OR fail* OR attack* OR insufficien*) NEAR/3 (heart OR cardia* OR myocard*)) OR (cerebrovascular* NEAR/3 accident*) OR cva OR stroke* OR ((brain OR cerebral) NEAR/3 (ischemi* OR ischaemi*)) OR mortalit*) OR (((diabet* OR dm) NEAR/3 (“type 2” OR type2 OR “type ii” OR “non insulin” OR noninsulin OR “adult onset” OR “slow onset” OR “maturity onset”)) OR T2DM OR dmt2 OR dm2 OR “T2- DM” OR “dm-t2” OR “dm-2” OR niddm OR “nid-dm” OR MODY))) NOT ((animal* OR rat OR rats OR mouse OR mice OR murine OR nonhuman* OR primate* OR hens) NOT (human* OR patient*))) AND DT = (article)

**Google scholar (first 200 results, out of 23′000)**

avena|oat|oats|oatmeal|granola|b-glucan|beta-glucan|“whole grain”|wholegrain|“whole grains”|wholegrains “cardiovascular|coronary|heart|myocardial|cardiac disease|failure|mortality”|diabetes|”type 2”|”non insulin”|noninsulin|NIDDM|T2D|mortality trial|study.
