# Supplementary material for: Oat Intake and Risk of Type 2 Diabetes, Cardiovascular Disease and All-Cause Mortality: A Systematic Review and Meta-Analysis
Source: Nutrients. 2021 Jul 26;13(8):2560. doi: 10.3390/nu13082560 (PMC8398256; doi:10.3390/nu13082560)
Supplement: Supplementary file 1 [file nutrients-13-02560-s001.zip › nutrients-1296950-supplementary.pdf]

Oat intake and risk of type 2 diabetes, cardiovascular disease and all-cause mortality: a systemic review and meta-analysis. Wehrli F

Supplementary Data

Table S1. Summary of the studies included in the systematic review.
Table S2. Extraction table for incidence of T2D.
Table S3. Extraction table for incidence of CVD.
A) Continuous exposure
B) Categorical exposure
Table S4. Extraction table for all-cause mortality.
Table S5. GRADE assessment. Does oat consumption influence the risk of T2D and CVD in the general population

Oat intake and risk of type 2 diabetes, cardiovascular disease and all-cause mortality: a systemic review and meta-analysis. Wehrli F

Supplementary Data

Table S1. Summary of the studies included in the systematic review.

Author, year	Funding	Country	Cohort name	Cohort size	Mean/median age at entry	% of males	Mean/median yeas of follow up	Baseline	Finish	Study design	Outcome definition	Dietary assessment method	Frequency of diet assessment	Control intervention	Number of events T2D	Number of events CVD	Number of events all-cause mortality	Adjustments	Stratified analysis by sex, age, BMI / tested interaction with sex, age, BMI	Quality score
Johnsen et al, 2014	Nordforsk and the Danish Cancer Society	Scandinavian	HELGA	119518 (w[1]: 80101, m[2]:39417)	W median (5-95th percentile) 51 (40-63)	33	NA	1992-1998	-	prospective	Vital status: National Central Population Registries, cause of death: National Cause of	semi-quantitative, country-specific and validated food-frequency questionnaire (FFQ)	baseline	-	24 w, 70 m	CHD[3]: 298 w, 858 m, stroke: 137 w, 14	7839	age, follow-up time, education, smoking, alcohol intake, BMI[4] and total energy intake	-	7
		Norway	Norwegian Women and Cancer Study	37111 (w)		0	11.1 median		2009					-			966		-	

		Sweden	Västerbotten Intervention Programme cohort (VIP)	25551 (w:13203, m:12348)	M median (5-95 percentile) 54(31-64)	48.3	14.2 median		2009		Death Registries			-		3 m	1367		NA	
		Denmark	Danish Diet Cancer and Health Study (DHC)	56865 (w:29787, m: 27069)		47.6	11.9 median		2008					-			5506		NA	
Lacoppidan et al, 2015	NordForsk (Centre of Excellence programme)	Denmark	DCH	55060	W all cases 3269 median (5 - 95th percentiles) 56 (50-64)	47.4	15 median	1995-1997	2011	prospective	T2D identified from the National Diabetes Registry	FFQ	baseline	-	7366	-	-	age, alcohol, smoking, education, participation in sports, intake of meat, total energy intake, waist circumference, body mass index	yes	8

	HELGA, the Danish Cancer Society.				M all cases 4097 n all cases 1570 median (5-95th percentiles) 55 (50-64)															
Helnaes et, 2016	Danish Cancer Society and Innovation Fund Denmark	Denmark	DHC	54871	all 25921 M median (5-95th percentiles) 55.0 (50.0-64.0)	47.2	13.6 median	1993-1997	2009	prospective	fatal MI[5] and nonfatal MI, (codes 410.0–410.99 and I21.0–I21.9) of the International Classification of Diseases (ICD). sudden Scardiac death	FFQ and 24-h dietary recalls	baseline	-	-	2329	-	School level, smoking, alcohol intake, participation in sports, w: menopausal status, hormone replacement therapy,intakes of fruit, vegetables, fish, red and processed meat, BMI, waist circumference, systolic blood pressure, self-reported hypertension, serum cholesterol,	yes	8
					M cases 1676 median age 57.0(51.0-64.0)															
					all 28950 W media															

					n age 56.0(50.0-64.0)<br>cases W 653 median age 59.0(51.0-64.0)						diagnosis (ICD 8: 427.27; or ICD 10: I46.0–I46.9).							hypercholesterolemia		
Hansen et al, 2017	Danish Council for Strategic Research, The Danish Cancer Society	Denmark	DHC	55338	all median (5-95th percentile) 56.1(50.7-64.2)<br>cases 2283 median (5-95th percentile) 58.7(51.0-64.7)	47.6	median 13.5	12.1993-05.1997	2009	prospective	Stroke: rapid onset disturbance of focal or global cerebral function lasting >24 hours or leading to death	FFQ	baseline	-	-	2283	-	Age, total energy intake, alcohol intake, physical activity, smoking and education, BMI-adjusted waist circumference, atrial fibrillation, hypertension, hypercholesterolemia and diabetes.	yes	8
Juan et al, 2017	National Cancer Instit	US	HPFS	42823	mean (SD) 53.2(9.5)	100	24	1986	2010	prospective	National Survey of Stroke,	Semi-quantitative FFQ	Baseline; every 4	-	-	908 strokes	-	Age, BMI, smoking status, alcohol intake, physical activity, family	-	7

	ute, National Heart, Lung and Blood Institute and National Institutes of Health (NIH)		NHS	71750	mean (SD) 50.1(7.1)	0	26	1984	2010	prospective	certificates, hospital records, autopsy records, telephone interview/ letter correspondence		years			1550 strokes		history of diabetes, cancer and heart disease, multivitamin use, hypertension, high cholesterol at baseline, total energy intake, modified alternative health eating index score. W: menopausal status, postmenopausal hormone use.	-	

Kyro et al, 2018	Swedish Research Council–Young Research Investigator Grant, the Danish Cancer Society, Innovation Fund Denmark	Denmark	DHC	55465	50-65	47.3	15	1995	2011	prospective	incident T2D	FFQ	baseline	-	7417	-	-	Age, educational level completed, Cambridge Physical Activity Index, smoking status, alcohol intake, intake of red and processed meat. W: menopausal status; use of menopausal hormone herapy	-	9

Wu et al, 2019	Academia Sinica	Taiwan	The Biosignature study	1663	no oat: 65.26 ±12.19 oat: 68.82 ±11.65	36.6	26.75 ±8.11 months	-	-	prospective	major cardiovascular events: cardiovascular death, non-fatal MI, non-fatal stroke, revascularization procedures including coronary intervention and bypass surgery	questionnare	baseline	not eating oat	-	175	-	Age, gender, hypertension, diabetes, smoking, ratio of waist to hip, medication, lipid, HsCRP, TNF-α.	-	6
Xu et al, 2019	University of Technology Syd-ney Facu	Australia	45 and Up Study	142503	45-64y 37,626 all cases<br>65-80y 24,203	-	6	2006-2009	2012-2015	longitudinal	heart disease, stroke, diabetes as response to "has a	brief questions	twice: baseline + 5 years later	-	5283	Heart disease 14148, str	-	Gender, marital status, education level, SEIFA, alcohol drinking, smoking and physical activity levels, BMI and for	stratified by age groups	5

	lty of Health seed funding, the Heart Foundation, New South Wales office for Health and Medical Research				all cases<br>>80 y 5967						doctor ever told you that you have”					oke 2911		fruit, vegetable, red and processed meat consumption.		
Lyskjaer et al, 2020	NA	Denmark	DHC	55095	median (5-95) 56.1(52.7–60.3)	47.6	13.4 median	12.1993-05.1997	30.11.2009	prospective	Nonfatal and fatal stroke: linkage to the Danish National Patient	192-item FFQ	baseline	eggs, yoghurt	-	2260	-	Total energy intake, sex, alcohol, physical activity, smoking,education level, BMI, mAHEI score, history of diabetes	by age	8

											Registe r.							mellitus, hypercholester olemia,hyperte nsion.		
Liu et al., 2000	NIH	US	NHS	755 21	38-63	0	10	198 4	1984	pros pecti ve	confir med type 2 diabete s	FFQ	4 year s	-	18 79	-	-	Age, body mass index; physical activity; cigarette smoking, alcohol intake; family history of diabetes in a first-degree relative; use of multivitamins or vitamin E supplements; total energy intake.	-	7

Olsen et al., 2011	The Danish Cancer Society and Nord Forsk	Denmark	DHC	57053	50-64	46.3	12	1997	2009	prospective	All-cause mortality	FFQ	-	-	-	-	4126	Age, time under study, smoking status, smoking duration, current tobacco consumption, time since cessation, alcohol intake, school education, participation in sports, time spent on sports per week, BMI, intake of red and processed meat, total energy intake	-	9
Liu et al., 1999	NIH	US	NHS	75521	38-63	0	10	1984	1.6.1994	prospective	incident CHD (fatal CHD, any non fatal MI)	FFQ	4 years	-	-	761	-	Age, body mass index, cigarette smoking, alcohol intake, parental or family history of MI before the age of 60 y, self-reported history of hypertension or hypercholestoremia, menopausal status , protein intake, aspirin	-	8

																		use, use of multiple vitamin or vitamin E supplements, vigorous activity, total energy intake, for fats, dietary fibers, folate, vitamin B-6, and vitamin E		
Wolk et al., 1999	NIH, Bethesda, Md.	US	NHS	68782	37-64	0	10	1984	1.6.1994	prospective	CHD (symptomatic nonfatal MI or fatal CHD)	FFQ	every 2 years	-	-	591	162	standard CHD risk factors	-	8
Hu et al., 2020	NIH	US	NHS	69139	38-63	0	mean 24	1984	2014	prospective	T2D, confirmed by at least one criteia of the National Diabetes Data Group	semiquantitative FFQ	2 years	-	18629	-	-	Age, ethnicity, smoking, alcohol intake, multivitamin use, physical activity, modified alternative healthy eating index, total energy, family history of diabetes, postmenopausal hormone use, oral contraceptive use, BMI	BMI, physical activity, smoking status, family history of diabetes	7
			NHS II	89120	27-42	0		1991	2017	prospective										
			HPFS	36525	40-75	100		1986	2016	prospective										

Supplementary Data

[1] – women
[2] – men
[3] – coronary heart disease
[4] - Body Mass Index
[5] - myocardial infarction

Table S2. Extraction table for incidence of T2D.

Author, year	Outcome definition	Cohort	Total number of events	Category Exposure										
				by group	cases	Total intake median, (5-95th percentile)	Total intake +/- SD	Not-adjusted		Age-adjusted model		Multivariable analysis		Stratified by age
								RR	95% CI	RR	95% CI	RR	95% CI	
Kyro et al, 2018	incident T2D	DHC	7417	m: Oat q1	1185	0 g/d	-	-	-	-	-	1		-
				m: Oat q4	777	>13 g/d	-	-	-	-	-	0.8	0,72-0,89	
				m: oatmealq1	2100	0 g/d	-	-	-	-	-	1		
				m: oatmealq4	686	>21 g/d	-	-	-	-	-	0.73	0,67-0,80	
				w: Oat q1	1099	0 g/d	-	-	-	-	-	1		
				w: Oat q4	695	>13 g/d	-	-	-	-	-	0.92	0,82-1,02	
				w: oatmealq1	1506	0 g/d	-	-	-	-	-	1		
				w: oatmealq4	446	>21 g/d	-	-	-	-	-	0.79	0,71-0,88	
Li et al., 2000	confirmed T2D	NHS	1879	Almost Never	-	-	-	-	-	1			-	-
				<40g/wk	-	-	-	-	-	0.97	0.88	1.07	-	
				80-160/wk	-	-	-	-	-	0.84	0.69	1.03	-	
				200–300/wk	-	-	-	-	-	0.61	0.32	1.15	-	
				≥40g/d	-	-	-	-	-	0.73	0.35	1.54	-	

X et al, 2019	diabetes in response to the question 'has a doctor ever told you that you have	45 and Up Study	5283	45-65 y	-	>0	-	0.72	0.65-0.76	-	-	0.85	0.76-0.92	yes
				65-80	-	>0	-	0.73	0.68-0.79	-	-	0.86	0.79-0.92	
				>80y	-	>0	-	0.78	0.69-0.85	-	-	0.86	0.74-0.98	
Kyro et al, 2018	incident T2D	DCH	7417	m: Oat q1	1185	0 g/d	-	-	-	-	-	1		-
				m: Oat q2	1107	0-1 g/d	-	-	-	-	-	0.93	0,86-1,01	
				m: Oat q3	1047	1-13 g/d	-	-	-	-	-	0.94	0,87-1,03	
				m: Oat q4	777	>13 g/d	-	-	-	-	-	0.8	0,72-0,89	
				m: oatmeal q1	2100	0 g/d	-	-	-	-	-	1		
				m: oatmeal q2	385	0-1 g/d	-	-	-	-	-	0.99	0,89-1,10	
				m: oatmeal q3	945	1-21 g/d	-	-	-	-	-	0.99	0,92-1,07	
				m: oatmeal q4	686	>21 g/d	-	-	-	-	-	0.73	0,67-0,80	
				w: Oat q1	1099	0 g/d	-	-	-	-	-	1		
				w: Oat q2	687	0-1 g/d	-	-	-	-	-	1.02	0,92-1,02	
				w: Oat q3	820	1-13 g/d	-	-	-	-	-	1.04	0,94-1,14	
				w: Oat q4	695	>13 g/d	-	-	-	-	-	0.92	0,82-1,02	
				w: oatmeal q1	1506	0 g/d	-	-	-	-	-	1		

				w: oatmealq2	394	0-1 g/d	-	-	-	-	-	1.05	0,94-1,18	
				w: oatmealq3	955	1-21 g/d	-	-	-	-	-	1.06	0,98-1,15	
				w: oatmealq4	446	>21 g/d	-	-	-	-	-	0.79	0,71-0,88	
Hu, et al., 2020	incident T2D	NHS	18629	w	9723	never or <40g/month	-	-	-	-	-	1	Ref	yes
		NHS II		w	6821	40g/month-40g/week						0.95	0.92-0.98	
		HPFS		m	2085	40g/week-160-240g/week						0.79	0.75-0.83	
		NHS			4555	never or <40g/month						1	Ref	
					2847	40g/month-40g/week						0.93	0.89-0.98	
					768	40g/week-160-240g/week						0.79	0.73-0.85	
		NHS II			3166	never or <40g/month						1	Ref	
					2961	40g/month-40g/week						1	0.95-1.05	
					945	40g/week-160-240g/week						0.83	0.77-0.89	
		HPFS			2002	never or <40g/month						1	Ref	
					1013	40g/month-40g/week						0.89	0.83-0.96	

					372	40g/week-160-240g/week						0.71	0.63-0.79	

Table S3. Extraction table for incidence of CVD.

A) Continuous exposure

Author, year	Outcome definition	Cohort	Total number of events	groups	cases	Total intake	Total intake +/- SD	continuous assessment	comparison	Not-adjusted		Age-adjusted model		Multivariable analysis		Stratified by age
										RR	95 % CI	RR	95 % CI	RR	95 % CI	
Juan et al, 2017	stroke	HPFS	908	-	-	40g/week	-	1 serving/week	-	-	-	0.79	0.57-1.09	0.93	0.67-1.28	-
		NHS	1550	-	-	40g/week	-		-	-	-	0.87	0.65-1.15	1.05	0.8-1.37	
Helnaes et al, 2016	MI	DHC	2329	m	all 25921	oatmeal medain (5- 95th percentile) 0.8(0.0-50.0)	13.1+/-20.1		-	-	-	-	-	-	-	-
						oats 1.0 (0.0-30.5)	8.3+/-12.4		-	-	-	-	-	-	-	-
					cases 1676	oatmeal 0.0(10.5-50.0)	10.5+/-18.5		-	-	-	0.81(per 25g	0.75-0.88	0.89 (per 25g	0.82-0.96	-

												increase)		increase)		
						oats 0.7 (0.0-30.5)	6.8+/-11.5		-	-	-	0.89 (per 10g increase)	0.85-0.93	0.96 (per 10g increase)	0.91-1.00	-
				w	all 28950	oatmeal 0,8(0.0-50.0)	11.5+/-18.6		-	-	-	-	-	-	-	-
						oats 1.1 (0.0-30.5)	7.5+/-11.6		-	-	-	-	-	-	-	-
					cases 653	oatmeal 0.0(0.0-50.0)	8.5+/-16.6		-	-	-	0.82 (per 25g increase)	0.71-0.95	0.92 (per 25g increase)	0.8-1.05	-
						oats 0.7(0.0-30.3)	5.7+/-10.3		-	-	-	0.85 (per 10g increase)	0.78-0.92	0.96 (per 10g increase)	0.88-1.05	-
Johnsen et al, 2014	CHD mortality	HELGA	1156	w	298	median 2	5-95th percentile 0-30	g/day	-	-	-	+follow-up years adjusted per doubling 0.95	0.9-1.0	0.95	0.9-1.01	
				m	858	median 2	5-95th percentile 0-31		-	-	-	0.98	0.95-1.01	0.98	0.95-1.02	

	fatal stroke		280	w	137	median 2	5-95th percentile 0-30		-	-	-	0.93	0.86-1.01	0.93	0.86-1.0	
				m	143	median 2	5-95th percentile 0-31		-	-	-	1.02	0.94-1.10	1.02	0.94-1.10	
Lyskjaer et al, 2020	Probable cases of nonfatal and fatal stroke by linkage to the Danish National Patient Register.	DHC	2260	-	all 55095	median 5g	5-95th percentile 0.0-150									calendar time, age at enrollment
				-	cases 2260	median 0 g	5-95th percentile 0.0-50		oatmeal for eggs			+calendar time 0.94	0.92-0.96	0.96	0.94-0.99	
									oatmeal for yogurt			+calendar time 0.97	0.95-0.99	0.98	0.98-1.01	
Xu et al, 2019	heart disease in response to: question "has a doctor ever told you that you have"	45 and Up Study	-	45-65 y	-	-	-	-	-	0.68	0.61-0.7	-	-	0.83	0.75-0.89	yes
			-	65-80	-	-	-	-	-	0.69	0.63-0.7	-	-	0.79	0.73-0.82	
			-	>80y	-	-	-	-	-	0.75	0.67-0.78	-	-	0.76	0.66-0.8	
	stroke as response to: "has a doctor ever told you that you have"		-	45-65 y	-	-	-		-	0.61	0.5-0.71	-	-	0.72	0.57-0.87	
			-	65-80	-	-	-		-	0.61	0.54-0.67	-	-	0.71	0.61-0.8	
			-	>80y	-	-	-		-	0.62	0.53-0.71	-	-	0.58	0.47-0.69	

Wu et al., 2019	cardiovascular death, non-fatal MI, non-fatal stroke	The Biosignature study	175	oat use	42	-	-		-	0.58	0,41 - 0,81	-	-	0.62	0,43 - 0,88	-
				no use	133	-	-		-							

B) Categorical exposure

Author, year	Outcome definition	Cohort	Total number of events	Group	Cases by group	Total intake median, (5th-95th percentile)	Age-adjusted model		Multivariable analysis	
							HR	95%CI	HR	95%CI
Juan et al, 2017	stroke	HPFS	908	424		< 40g/month	1		1	
				246		40g/month-40g/week	0.92	0.78-1.07	0.95	0.81-1.11
				238		> 40g/week	0.83	0.7-0.97	0.9	0.76-1.06
		NHS	1550	543		< 40g/month	1		1	
				603		40g/month-40g/week	1.04	0.92-1.17	1.1	0.97-1.24
				404		> 40g/week	0.98	0.86-1.11	1.1	0.96-1.26
Hansen et al, 2017	stroke	DHC	2283	-	1765	<21 g/day	1		1	
				-	518	>=21 g/day	0.76	0.69-0.84	0.85	0.77-0.95
Johnsen et al, 2014	CHD mortality	HELGA	298	w: CHD	298	q 1:0 g/day	-	-	1	
						q 2:0.4	-	-	0.74	0.52-1.05
	fatal stroke		0			q 3:3	-	-	0.85	0.59-1.21
						q 4:14	-	-	0.66	0.45-0.96
				m: CHD	418	q 1:0	-	-	1	
						q 2:0.5	-	-	1.01	0.83-1.23
						q 3:4	-	-	1.01	0.8-1.28
						q 4:30	-	-	0.82	0.65-1.03
					298	q 1:0	-	-	1	

				w: stroke		q 2:0.4	-	-	1.03	0.64-1.67
						q 3:3	-	-	0.66	0.38-1.16
						q 4:20	-	-	0.63	0.34-1.15
				m: stroke	418	q 1:0	-	-	1	
						q 2:0.5	-	-	0.67	0.42-1.07
						q 3:5	-	-	1.03	0.6-1.76
						q 4:30	-	-	0.71	0.41-1.21

Table S4. Extraction table for all-cause mortality.

		Category Exposure								Continuous exposure				
Author, year	Cohort	sex	cases by group	Total intake median	Total intake 5th–95th percentiles	Age- and follow up time adjusted		Multivariable analysis		sex	cases	Total intake median in g/day	Multivariable analysis	
						RR	95%CI	RR	95%CI				HR	95%CI
Johnsen et al, 2014	HELGA	w	3658	2	0-30	per doubling effect 0.98	0.96-0.99	0.98	0.96-0.99	w	3658	q[1]1: 0	1	
		m	4181	2	0-31	0.98	0.97-1.0	0.98	0.97-1.0			q2: 0.4	0.85	0.78-0.93
	Norwegian Women and Cancer Study	w		1	0-22							q3: 4	0.74	0.67-0.82
	VIP	w		5	0.3-25							q4: 19	0.78	0.7-0.87
		m		5	0.4-31					m	4181	q1: 0	1	
	DHC	w		1	0-30							q2: 0.4	0.87	0.8-0.95
		m	7839	0.9	0-30							q3: 3	0.85	0.77-0.94
												q4: 30	0.76	0.69-0.85

[1] - quartile

Table S5. GRADE assessment. Does oat consumption influence the risk of T2D and CVD in the general population.

Quality assessment								No of study subjects		Relative (95% CI)	Quality	Importance
	Design	No of studies	Risk of bias	Inconsistency	Indirectness	Imprecision	Other considerations	Total	Events			
**Outcome 1**												
T2D (low vs high)	Observational[1]	4	No concern[2]	No concern[3]	No concern[4]	No concern[5]	No evident publication bias[6]; no upgrading[7]	250249	26046	0.78 (0.74-0.82)	low	Critical
**Outcome 2**												
T2D (consumer vs. non consumer)	Observational[1]	3	-1[8]	-1[9]	No concern[10]	-1[11]	No evident publication bias[12]; no upgrading[13]	273489	14579	0.86 (0.72-1.03)	very low	Critical
**Outcome 3**												
CHD and MI	Observational[1]	2	-1[14]	-1[15]	No concern[16]	-1[17]	No evident publication bias[18]; no upgrading[19]	197374	16477	0.81 (0.61-1.08)	very low	Critical
**Outcome 4**												
Stroke	Observational[1]	4	No concern[20]	-1[21]	No concern[22]	-1[23]	No evident publication bias[6]; no upgrading[24]	312171	7629	0.79 (0.59-1.07)	very low	Critical
**Outcome 5**												
Composite CVD	Observational[1]	2	-1[25]	-1[26]	No concern[27]	-1[28]	No evident publication bias[18]; no upgrading[29]	121181	1611	0.73 (0.5-1.07)	very low	Critical

Oat intake and risk of type 2 diabetes, cardiovascular disease and all-cause mortality: a systemic review and meta-analysis. Wehrli F

Supplementary Data

[1] All the studies were prospective cohort studies
[2] All the studies are of high quality, so we do not think bias is a concern in this outcome.
[3] I^2 =47% (moderate heterogeneity), p = 0.129; no wide variation of point estimate across studies; confidence intervals are mostly overlapping
[4] Two studies included only women, one study included only men, one study included men and women; three studies from North America, one study is from Europe. The study results can be generalized to the general population.
[5] Large number of participants, including cases with T2D (250,249, therefrom 26,046 T2D cases); effect size statistically significant (95% CI does not include 1).
[6] Due to a small number of studies (n=4), publication bias was not formally assessed.
[7] No upgrade due to small effect size, accounted for confounders (age, smoking, alcohol intake, postmenopausal hormone use), however, other important confounders were not taken into account (residual confounding); no evidence for dose-response relationship.
[8] Assessed by Newcastle-Ottawa Scale one study was of good quality, two of fair quality, therefore bias is a concern in this outcome.
[9] High heterogeneity: I^2= 97.6%, p=0.000 (considerate heterogeneity); point estimates demonstrate high variability, one study has no overlap of 95% CI with others.
[10] One study was conducted in Northern America, one in Europe, one in Australia, therefore we think the results can be generalized to the general population.
[11] Large number of participants, including cases with T2D (273,489, therefrom 14,579 T2D cases); effect size is not statistically significant (95% CI includes 1).
[12] Due to a small number of studies (n=3), publication bias was not formally assessed.
[13] No upgrade due to small effect size, accounted for confounders (alcohol intake, smoking), however, other important confounders were not taken into account (residual confounding); no evidence for dose-response relationship.
[14] Assessed by Newcastle-Ottawa Scale one study was of good quality, one of fair quality, therefore, bias is a concern in this outcome.
[15] I^2 =99.2%, p=0.000 (considerate heterogeneity); point estimates demonstrate high variability; 95% confidence intervals do not overlap.
[16] One study conducted in Europe and one from Australia, the study results can be generalized to the general population, both sexes were assessed, no difference between genders, when reported separately.
[17] Large number of participants, including cases with CHD and MI (197,374, therefrom 16,477 CHD and MI cases); effect size is not statistically significant (95% CI includes 1).
[18] Due to a small number of studies (n=2), publication bias was not formally assessed.
[19] No upgrade due to small effect size, accounted for confounders (educational status, smoking, alcohol intake, physical activity, fruit and meat intake), however, other important confounders were not taken into account (residual confounding); no evidence for dose-response relationship.
[20]Assessed by Newcastle-Ottawa Scale three studies out of four were of fair quality, one of good quality, therefore we not think bias is a concern in this outcome.
[21] High heterogeneity: I^2= 97.2%, p=0.000 (considerate heterogeneity); point estimates demonstrate high variability, one study has no overlap of 95% CI with others.
[22] Two studies were conducted in Northern America, one in Europe and one in Australia, therefore we think the results can be generalized to the general population.
[23] Large number of participants, including cases with stroke (197,374, therefrom 7,629 stroke cases); effect size is not statistically significant (95% CI includes 1).
[24] No upgrade due to small effect size, accounted for confounders (BMI, physical activity, alcohol intake, smoking), however, other important confounders were not taken into account (residual confounding); no evidence for dose-response relationship.
[25] Assessed by Newcastle-Ottawa Scale both studies were of fair quality, therefore, bias is a concern in this outcome.
[26] I^2 =79.1%, p=0.029 (considerate heterogeneity); point estimates demonstrate high variability; 95% confidence intervals barely overlap.
[27] One study conducted in Europe and one is from Taiwan, the study results can be generalized to the general population, both sexes were assessed, no difference between genders, when reported separately.
[28] Large number of participants, including cases with CVD (121,181, therefrom 1,611 CVD cases); effect size is not statistically significant (95% CI includes 1).

[29] No upgrade due to small effect size, accounted for confounders (age and smoking), however, other important confounders were not taken into account (residual confounding); no evidence for dose-response relationship.
